# “Breaking” the Emergency Department: Does the Culture of Emergency Medicine Present a Barrier to Self-Care?

**DOI:** 10.5811/westjem.2019.10.44584

**Published:** 2020-02-21

**Authors:** James O’Shea, Salwar Vu, Jeffrey Siegelman, Sheryl Heron, Michelle Lall

**Affiliations:** Emory University, Department of Emergency Medicine, Atlanta, Georgia

## Abstract

**Introduction:**

Our goal was to critically examine emergency physician’s (EP) beliefs about taking breaks for self-care on shift. Our operational definition of a break for self-care included time not engaging in direct patient care, eating, drinking, using the bathroom, or leaving a clinical area for a mental break. Using focus groups, the study aimed to accomplish the following: 1) identify barriers to why residents and faculty at our academic center may not take breaks in the emergency department; 2) generate hypotheses for empirical testing; and 3) generate solutions to include in a departmental breaks initiative.

**Methods:**

We convened eight focus groups comprised separately of resident and faculty physicians. Group discussion was guided by eight questions representing a priori themes. The groups were recorded for transcription and subjected to a “cut-and-sort” process. Six themes were identified by consensus after independent review by three of the co-authors, which were confirmed by participant validation.

**Results:**

We identified six themes that represented the pooled outcomes of both resident and faculty focus groups: 1) Physiological needs affect clinical performance, 2) EPs share beliefs around taking breaks that center on productivity, patient safety and the dichotomy of strength/weakness, 3) when taking breaks EPs fear worst-case scenarios, 4) breaking is a learned skill, 5) culture change is needed to allow EPs to engage in self-care; and 6) a flexible, individualized approach to breaking is necessary. Our central finding was that productivity and patient safety are of key importance to EPs when considering whether to take a break for self-care. We identified a dichotomy with the concept of strength related to productivity/patient safety, and the concept of weakness related to self-care.

**Conclusion:**

The current practice culture of emergency medicine and the organization of our unique work environment may present barriers to physicians attempting to engage in self-care.

## INTRODUCTION

“We have all felt it. The fatigue. The hunger. The hazy fog that ensues 11 hours into our shift.^”1^

Many industries recognize the connection between rest breaks on shift and the optimization of performance and the reduction of errors.^2^–^4^ Shift-workers in particular have an increased risk of occupational injury, disability and poor health.^5^ There is evidence from the healthcare industry that fatigued shift-workers make more medical errors.^6^–^8^ The recognition of this link between fatigue and error has led to a focus on resident duty-hour restrictions to promote healthcare safety and physician wellness.^9^ Such measures address rest off shift but do not address the possible need for self-care while on shift.

Emergency physicians (EP) work fewer hours on average than many other medical specialties, theoretically affording more time off shift for rest. However, emergency medicine (EM) clinical shifts are fast-paced, and there is a high density of cognitive work and decision-making with providers suffering high burnout rates.^10^ It could be argued that in terms of rest, EM is a special case, with a potential need for on-shift breaks to account for the high pace. In addition, duty-hour restrictions focus on physical rest including sleep, but cognitive functioning may also be significantly impacted by immediate physiological needs such as hunger, thirst, and the need to use the bathroom.

There is a paucity of literature on EP breaks for self-care during shifts. We identified a single study during our literature review on the effects of an on-shift break on EPs’ clinical performance. This study found that EPs reported significantly less tiredness at the end of their shift if they had taken a break.^11^ Intriguingly, for the cohort that took breaks, there were significant improvements in time-to-provider metrics for triage category two and three patients, as well as significant improvements in “time to admission.” This study also points to a possible link between a more efficient, rested physician and improved emergency department (ED) flow metrics. Given the increasing demand on EPs to improve their practice efficiency and meet key performance metrics,^12^ factors with a potential influence on worker efficiency such as breaks should be empirically investigated.

To begin to investigate the concept of taking rest breaks for self-care while working in the ED, we developed and conducted focus groups to qualitatively examine this specific aspect of our professional practice culture. Our objective was to critically examine our EPs’ existing cultural beliefs about taking breaks for self-care on shift in the ED using separate focus groups comprised of resident and attending physicians. It was our hope that in doing so, we could inform a departmental breaks initiative and generate hypotheses for empirical testing.

## METHODS

### Study Design

During two separate retreats for both residents and faculty, we conducted a total of eight focus groups of approximately 15 participants per group. The study was given an institutional review board exemption after initial board review.

### Study Setting

Within a single, large, academic institution that incorporates five different EDs representing a spectrum of settings from academic to community, 116 EPs took part in focus groups. This included 56 attending physicians who chose to attend a department-wide faculty retreat and 60 resident physicians who participated during an annual mandatory residency retreat.

### Study Protocol

Residents were divided into four groups, and there were four faculty moderators who guided the discussions. Moderators received a brief training in focus group dynamics and used eight structured questions to guide the focus groups over the course of an hour. These eight questions were developed based on a priori themes identified by consensus among the investigators. The moderators were faculty known to the resident and attending physicians. We repeated this process with four additional focus groups conducted during a faculty wellness retreat. The faculty and resident focus groups were digitally recorded and transcribed for qualitative analysis.

**Population Health Research Capsule**

What do we already know about this issue?

While many industries recognize the connection between rest breaks, wellbeing and error reduction, there are few published studies on break-taking in emergency medicine (EM).

What was the research question?

Are there professional cultural beliefs that might be a barrier to physicians at our institution taking breaks on shift?

What was the major finding of the study?

EM culture shows a central dichotomy with strength related to productivity/patient safety, and weakness related to self-care.

How does this improve population health?

Our findings can promote policies that support on-shift cognitive function and physician health, which may result in improved performance and better health outcomes for patients.

### Data Analysis

Post-transcription coding was completed by digital pawing and the cut-and-sort method. Transcripts were analyzed separately by the principal investigator and two peers. Team analysis then resulted in consensus on six main themes in both faculty and resident cohorts, which were confirmed by participant validation.

## RESULTS

The initial focus group was conducted exclusively with 60 resident physicians and generated six themes. The second focus group was conducted with 56 EM faculty physicians who work at five different sites that range from academic to community settings. There was a high degree of convergence between themes, such that the respondents could be pooled to yield six overarching themes. Underlying differences between the groups existed but were subtle. For example, the belief by many respondents that permission was needed to feel “allowed” to engage in breaks was a common theme. However, residents needed explicit permission from senior residents and attending physicians to engage in self-care, whereas some attending physicians needed permission from colleagues and other staff.

Both attending and resident groups ranked the importance of dealing with particular physiological needs in the same order and at similar frequencies (Table 1) and had similar responses to the ideal length of a break (Table 2).

We report here six overarching themes that captured the responses of both groups.

### Focus Group Themes

Table 3 lists the themes that were identified, and key quotations supporting each theme are presented in Table 4.

1. EPs frequently experience basic physiological needs on shift, which can negatively affect cognitive function and emotional self-regulation.

‘I get cognitively fatigued, I guess, six or seven hours in. And panicked that I’m not going to last… my brain is just falling apart.’

‘I realize my sign-outs are terrible and I can’t focus and that’s when I know I am hungry.’

‘That’s how I know it is time to take my break. When I walk into the patient’s room and the rage builds instantly. I have no patience, so I go eat for five minutes.’

The physicians and residents in our study readily identified with the experience of hunger negatively affecting their performance on shift. There was a belief that EPs’ decision-making and ability to moderate emotional reactions to work-related stressors were also affected. The responses indicated that these negative effects could be positively impacted by caloric intake and cognitive breaks.

2. EPs share beliefs around break-taking that amount to “culture.”

‘I think that, to get to where we are we’ve had to be very strong. And you don’t want anyone to see your weak side, which might mean needing to go to the bathroom, or needing to eat.’

‘..because I always have my water with me; you will never see me without it and I will have a snack-bar in my pocket in case I am hungry; by taking the time off (to have a break) you get judged in your career.’

**Figure 1:**
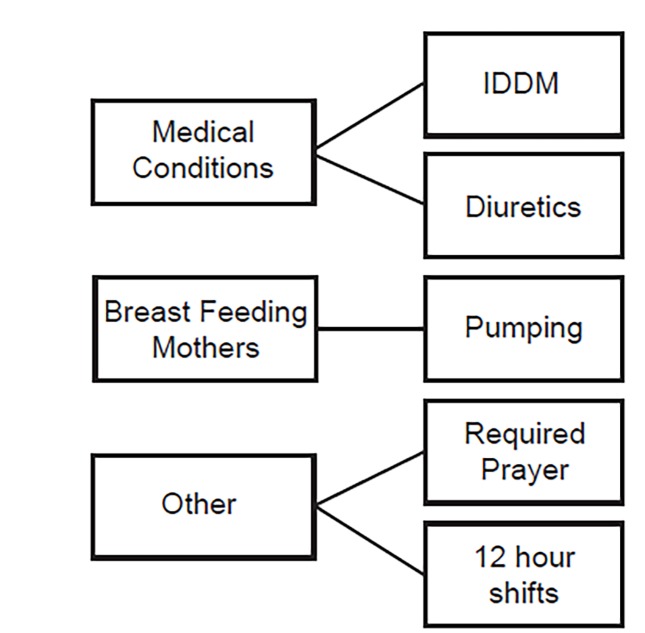
There were several permitted exceptions to the strength/weakness dichotomy where participants overtly agreed with break-taking behavior.

The responses suggest that there is a set of values and conventions associated with self-care in EM professional culture. The responses point to the implication that productivity/patient safety is strength, and self-care is weakness. The groups identified several exceptions to this culture, as shown in Figure 1.

3. When considering the impact of breaks for self-care on patient safety, EPs frequently imagine worst-case scenarios. However, reports of direct personal experience of negative outcomes are rare.

‘I don’t think I’ve ever seen anything bad happen, but I’ve certainly sat through 15 years of M&Ms, where people who have waited and have died in the bathroom in the waiting room. So, I know it’s a real thing. It’s a hard thing to quantify.’

‘It’s the difference between perception and reality…the reality is that the events are few and far between and probably could be mitigated with a little planning. But definitely the perception is… you know... you got sick people.’

‘Let me tell you how I learned that it’s ok to take breaks. When I was breastfeeding. And guess what, when I came back, all was the same as when I left. Nothing bad happened.’

There is a clear concern among EPs that taking a break will impact patient care. This concern took two forms. First was a concern that patients would become critically unwell in their absence and without their knowledge. Second, they worried that throughput would drop and the flow of patients from the waiting room would slow, thereby keeping an unrecognized unwell patient in the waiting room. This implies a connection between EP break-taking, productivity, and patient safety. If you take a break, you will see fewer patients, those unseen patients may decompensate during the delay, and it will be your fault. When talking about the potential risks to their patients, study participants tended to frequently imagine worst-case scenarios. Among the 116 EPs involved in the focus group process, near-misses were reported but there were no direct personal experiences of serious negative patient outcomes.

4. Taking a break is a skill that requires practice and safety-oriented communication strategies.

‘It’s almost like leaving your child with a babysitter. It’s having the experience of knowing when it’s appropriate to go. Do you know what I mean? There’s a skill to taking a break, knowing if this is appropriate timing. Maybe, even if it was the culture that we take break, maybe still 1/3 or 1/4 of shifts we wouldn’t take breaks.’

‘…but can’t you be within a vicinity where they can say, “Hey doc, I need you, this patient’s taking a turn for the worse.”? Yea, be somewhere where they can call you.’’

‘I guess if you’re gone, as long as and people know that you’re gone and where to find you…like if you’re in the resident room, so you’re easily accessible.’

The practice culture of EM requires the acquisition of a well-defined set of learned skills. Group participants responded to break-taking as a learned skill that required timing, intuition, and situational awareness. There was a recognition that this skill required practice in training, until a sense of timing evolved. This could be supported in training by examination of teachable moments where timing was suboptimal. In addition, there was a sense that communication strategies could be learned in order to support safe break-taking. These strategies include explaining to the nursing and ancillary staff what you are doing, where you will be, and having a means of being contacted. The ideal location made both physical absence, to protect the EP from unnecessary interruption, and a timely return to the bedside possible.

5. A new cultural norm in EM would require negotiated agreement with peers and other staff in order to give participants permission to engage in self-care.

‘Help us understand that it’s normal, and it’s ok, and you don’t have to do anything targeted or specific. You just need to walk away from the space, get some clarity and re-focus, and then you can continue to do your work.’

I think it needs to be part of the culture. And that’s the only time someone’s come to me and said, “Hey, I’m gonna be gone for a minute,” is when they’re going to breastfeed. That’s when it’s acceptable. But otherwise no--

‘Well, I’ve done it (taken a break), when the Chair came in and she said, ‘good for you’ and we sat down and talked and ate together and I think if the Chair of the department sat down with me to talk with me then it is ok. And told me ‘Good for you,’ that’s what she said!’

Changing an accepted cultural norm requires agreement and permission to become accepted. Group participants who were early adopters or pioneers risked being viewed as outside the prevailing culture by making an individual decision to engage in a behavior that appeared non-standard. Both resident and faculty groups included a minority of individuals with a “pioneer” or “early adopter” mindset, and a larger majority that needed to negotiate agreed cultural change and permission before engaging in self-care.

6. Taking a break requires strategies that are flexible and individualized.

‘You gotta chart, I mean you can chomp and chart.’

‘I bring a smoothie and have it on my desk...so if I end up feeling shaky or weird I can end up drinking the smoothie’.

‘I take micro breaks. I’m a micro breaker. And maybe if you add it all together it equals a half-hour break, but I mean, if I have to pee, I’ll find a good time, go pee for three minutes. Go get coffee for seven minutes, black out on my desk for three minutes.’

What is defined as a break varies across shifts and between individuals, in terms of what is occurring clinically and what the physician needs to have occur to support their optimal physical and cognitive function. For many EPs, meeting their physiological needs on shift requires a variety of strategies, breaks of varied durations, and “formal” vs “informal” eating depending on the circumstances.

## DISCUSSION

The current study points to a professional culture in emergency medicine in which an EP who cannot go for 8–12 hours without attending to their basic physiological needs during a period of intense cognitive, physical and emotional work runs the risk of being perceived as “weak.” While this study was performed on EPs, the strength/weakness dichotomy may well be an issue more broadly in medical culture. As an occupational group, medical doctors are often viewed as special cases. Few other professions are tasked with sustaining such a high level of productivity, quality, and safety over a working week that can be exceptionally long. This special treatment often extends to an expectation that medical doctors can maintain this productivity, quality, and safety without a formal break to attend to the “housekeeping” that comes with having a body and a mind.

There have been sporadic efforts to introduce mandated rest breaks in the field of medicine. The Australian Medical Association’s National Code of Practice makes provision for a mandatory 30-minute meal break, which was proposed in order to reduce fatigue during shifts.^13^ In the European Union, a law entitled the “Working Time Directive” mandates 20 minutes of rest after every six hours of work, with no routine exemption for physicians and significant financial fines imposed on employers for infringements.^14^

The American College of Emergency Physicians recently collaborated with the Joint Commission (JCAHO) to challenge a common misconception among EPs that they are prohibited by JCAHO from eating or drinking at their work stations.^17^ This clarification was sought in recognition that “not being able to have a drink or eat in the ED can significantly impact both the physical well-being of emergency physicians and their decision-making ability, and therefore risks impacting not just their own health, but that of their patients too.”^18^

In terms of health policy, we believe there should be a national conversation about embedding rest breaks into the working lives of doctors. This would require national and organizational change but also a shift away from the professional culture identified in this study of academic EPs that equates self-care with weakness. In the recent Blue Ridge report on wellness in academic centers, there was explicit recognition that attending physicians’ behavior is of key importance in modeling wellness behaviors for residents as they train and acquire the skills necessary to work in the ED.^17^ This is certainly true in medical training generally, and implies a significant responsibility on the part of attending physicians to model self-care behaviors to residents that will ensure sustained wellness and productivity over a long career. Our findings indicate that rather than training residents how to care for themselves, we are training them to ignore basic physiological needs, which may impact not only their wellness but also clinical decision-making and patient safety.^18^,^19^

Our study findings underline the fact that productivity and patient safety are of fundamental importance to physicians. In other manufacturing industries, taking breaks decreases occupational injuries, presumably through improved cognitive function.^20^ In our groups there was debate among study subjects over whether a break might potentially improve cognitive function and thus safety and productivity. This gain could potentially offset the “lost” time spent engaging in self-care or perhaps taking a break would just negatively impact productivity. Indeed, there was a general sense that while subjects had opinions on this, no one objectively knew the actual impact of their own behavior on their productivity, and there was a strong interest among our subjects in this being studied empirically.

Our study respondents suggested that clear communication strategies could ensure safety for patients without requiring the addition of personnel coverage. Such strategies would involve having a location in the immediate vicinity of the clinical work area but out of the line of sight of personnel who may make non-critical requests or other interruptions. It would also involve clear communication with the charge nurse and other relevant colleagues. More controversial was the idea of keeping a phone or radio to maintain situational awareness and remain contactable. Some EPs felt this made the break possible by ensuring safety, while others felt that a high volume of un-triaged calls could make a break untenable. This would suggest that how these devices are used locally can make them either an “alleviating or aggravating” factor.

Our study shows that having a break imposed on an EP by a superordinate issue such as a medical condition or the desire to pump breast milk for one’s child can transcend professional cultural judgment and allow an opportunity for EPs to realize that self-care may not necessarily be anathema to patient safety. Within the general cohort of EPs, breast-feeding mothers formed a distinct subgroup that contributed significantly to the discussion. Breast-feeding mothers have to stop on shift to pump breast milk, and this seems to be a “culturally allowed” exception in our study cohort, in addition to those with medical conditions such as insulin-dependent diabetes or clinicians working 12-hour shifts. EPs in this cohort unequivocally supported breast-feeding mothers and their need to have a break. The EPs who did have the experience of regularly taking a break to pump reported that their previous “worst-case scenario” assessments of the effect of their brief absence on patient safety in hindsight seemed to be inaccurate.

EPs recognized the need to come prepared for a break and that sources of nutrition and hydration ideally need to be prepared and brought to work. Agreement that taking a break is acceptable must be secured locally within individual institutions or work units, and this local agreement could be aided by a broader shift in our national professional culture. Clear communication with other staff needs to occur to ensure patient safety and also to protect the EP from non-critical interruptions. The ability to return in a timely fashion in the event of an emergency is vital, particularly where there is no covering physician. Lastly, the core of the skill is a sense of timing, knowing when it is appropriate to leave given the current status of the ED and the disposition of one’s patients. The goal vision arising from the group participants is that of the physician as an organizationally supported and culturally empowered decision-maker equipped with the skills and logistics required to care for themselves and, therefore, their patients.

## LIMITATIONS

This was a single-center study in an academic environment, and the findings are specific to an urban, academic setting. The focus group facilitators were faculty members who were known to the study participants, and this may have had an impact on responses. The current study did not include an empiric data-gathering phase that would have created a taxonomy of current break-taking behavior and instead started with the a priori assumption, based on the research team’s experience, that many EPs at our center do not take breaks on shift. While there was a mix of academic and community-based EPs, faculty participants were more likely to work in an academic setting with residents, which allows the opportunity for coverage. In addition, while study participants worked at a variety of sites, they were all under the umbrella of a single organization, with its own professional culture and, thus, the results may not be generalizable outside this organization or to a community-based practice setting.

## FUTURE DIRECTIONS

EPs are primarily concerned with the safety of their patients and with maintaining their productivity in order to provide efficient timely care for others. We believe that a national survey of current break-taking behavior among EPs would delineate the important issues and help guide further study and the design of educational interventions. It is likely that the need for a break and its effect on cognitive function and decision-making is variable across EPs, but we lack national data to understand this variability and the extent of current self-care behavior. Our qualitative study data also suggests a number of testable hypotheses for future empirical or mixed methods research. Showing benefit beyond productivity is also important. Does eating on shift enhance the EP’s important transition to home life post-shift? It may be that the deleterious effects of not taking a break are seen at home rather than on shift.

We suggest that concerns about patient safety are of such central importance to EPs that no cultural change will be possible without data showing that EPs who engage in well-planned, self-care breaks with built-in safety strategies can take breaks without affecting the safety of their patients. Also, empirical research into this question could help delineate the types of safety strategies that work in different practice environments. Such data could also help change the existing cultural belief that self-care and patient safety are opposed and aid in the education of residents in this self-care skill.

## CONCLUSION

Training that incorporates self-care as a means of optimizing cognitive performance and emotional self-regulation is more critical than ever given the current high rates of physician burnout and concerns for patient safety. To deliver this training, EM must first acknowledge where we are in terms of our professional culture. While major strides have been made toward placing wellness at the center of EM training, there remains a persistent and uninvestigated professional culture of poor self-care on shift that is ready for examination and change.

## Figures and Tables

**Table 1:** Frequency in text – self-care needs of emergency medicine attendings and residents.

	Hunger	Thirst	Bathroom	Cognitive Break
Attendings	64.4%	18.2%	13.4%	3.8%
Residents	53%	23%	12%	12%

**Table 2:** Frequency in text – ideal break length.

	>20 mins	10–20 mins	< 10 mins
Attendings	9.9%	87.9%	12.1%
Residents	7%	79.4%	13.6%

**Table 3:** Themes identified in the focus group discussion.

1	Emergency physicians (EP) frequently experience basic physiological needs such as hunger on shift, which can negatively affect cognitive function and emotional self-regulation.
2	EPs share beliefs about break-taking that amount to “culture.” These beliefs center around productivity, patient safety, and the dichotomy of strength/weakness.
3	When considering the impact of taking a break on patient safety, EPs frequently imagine worst-case scenarios. However, reports of direct personal experience involving negative outcomes were uncommon.
4	Break-taking is a skill that requires practice, appropriate timing, and safety-oriented communication strategies.
5	A new cultural norm requires negotiated agreement with peers and other staff in order to give participants permission to engage in self-care.
6	The ability to engage in break-taking behavior requires strategies that are flexible and individualized.

**Table 4:** Key quotations identified in the focus group discussion.

Theme	Sub-theme	Key quotations
Emergency physicians frequently experience basic physiological needs such as hunger on shift that can negatively affect cognitive function and emotional self-regulation.	Cognitive Function	“I get cognitively fatigued, I guess, 6 or 7 hours in. And panicked that I’m not going to last… my brain is just falling apart..” “My brain can’t focus, prioritize and or multitask.” “I realize my sign-outs are terrible and I can’t focus and that’s when I know I am hungry.”
	Emotional Self-Regulation	“That’s how I know it is time to take my break. When I walk into the patient’s room and the rage builds instantly. I have no patience so I go eat for five minutes.” “You lose patience with people...how you react to consultants or patients...I get outright cranky.”
Culture of Break-Taking	Culture	“..you are soft, you guys are taking wellness breaks, back in our day we never had this and are tougher doctors for it, made me feel like crap and not want to take breaks.” “..because I always have my water with me; you will never see me without it and I will have a bar in my pocket in case I am hungry; by taking the time off (to have a break) you get judged in your career.” “I got food and I started to eat and I felt like I was doing something wrong. Look at me, oh my God! What if someone sees me! I felt like I was doing something so wrong, eating for those 15 minutes.”
	Strength/Weakness Dichotomy	“I think you feel guilty, almost weak for needing to stop. In fact, now that I’m older, I feel weak that I have to go to the bathroom twice during a shift. I used to be able to go the whole 12 hours without going.” “No, no, no. I know that anyone would be willing to cover me, it’s more that feeling of weakness.”
Fear of Worst-Case Scenarios	Worst-Case Scenariors	“I don’t think I’ve ever seen anything bad happen, but I’ve certainly sat through 15 years of M&Ms, where people who have waited and have died in the bathroom in the waiting room. So I know it’s a real thing. It’s a hard thing to quantify.” “Actually, I don’t take breaks because of that. There’s not really anybody to cover you when you have 13 sick and dying patients.” “I’ve had patients who’ve deteriorated while I’ve been in the bathroom. The nurse is looking for you, you know… actually the other day a resident was looking for me and I was coming out of the bathroom, and they were like “We need you in room…”; but luckily I wasn’t gone long enough. I guess if you’re gone, as long as and people know that you’re gone and where to find you…so you’re easily accessible.”
	Direct Experience	“Let me tell you how I learned that it’s ok to take breaks. When I was breastfeeding. And guess what, when I came back, all was the same as when I left. Nothing bad happened.” “It’s the difference between perception and reality…the reality is that the events are few and far between and probably could be mitigated with a little planning. But definitely the perception is… you know... you got sick people.” “I was getting food in the cafeteria with my radio when they called that something was coming in and I got there before the patient but I didn’t like that feeling. Nobody said anything but I didn’t like the feeling of being unprepared. It was one of those 2 minutes CPR patients.”
Break as Learned Skill	A Practiced Skill	“It’s almost like leaving your child with a babysitter. It’s having the experience of knowing when it’s appropriate to go. Do you know what I mean? There’s a skill to taking a break, knowing if this is appropriate timing. Maybe, even if it was the culture that we take a break, maybe still 1/3 or 1/4 of shifts we wouldn’t take breaks. Maybe the point is that this is something that could be taught.” “It is a learned skill. There was a point in time where I felt I couldn’t leave. Then there was a point in time where it felt like I could go stand in the corner for 5 minutes. And now I’m fine leaving for 25 minutes.” “You know what’s going on, you still control the flow, and you made a conscious decision at this time that it’s safe and reasonable to take a break. As opposed to, “Oh it’s 12, I have no idea what’s going on, but I’m leaving.”
	Safety-Oriented Communication Strategies	“…but can’t you be within a vicinity where they can say, “Hey doc, I need you, this patient’s taking a turn for the worse.”? Yea, be somewhere where they can call you.” “I guess if you’re gone, as long as and people know that you’re gone and where to find you…like if you’re in the resident room, so you’re easily accessible.” “It was sort of very clear; you would let your attending know. It wasn’t so much that you would sign out to another resident, you would just let your attending know, like ‘Here are my patients,’ tell them whoever you were worried about” “I think it’s good to have the phones with you, because that way you know if something critical is coming in, they need you and you can stop whatever you’re doing no matter how important it is and you can rush back…if they need more help. Whereas if you don’t have a phone you’re completely unaware...what if people need you, what if they need more manpower.”
Permission Required		“Help us understand that it’s normal, and it’s ok, and you don’t have to do anything targeted or specific. You just need to walk away from the space, get some clarify and re-focus, and then you can continue to do your work.” “I think it needs to be part of the culture. And that’s the only time someone’s come to me and said, “hey I’m gonna be gone for a minute,” is when they’re going to breastfeed. That’s when it’s acceptable. But otherwise no—” “Well, I’ve done it (taken a break), when the Chair came in and she said ‘good for you’ and we sat down and talked and ate together and I think if the Chair of the Department sat down with me to talk with me then it is ok. And told me ‘Good for you’ that’s what she said!”
Execution Requires Flexible, Individualized Approach	Strategies	“You gotta chart, I mean you can chomp and chart.” “I never bring in anything that would require a fork. It’s always a sandwich I can shove down in 2 minutes or less.” “I bring a smoothie and have it on my desk...so if I end up feeling shaky or weird I can end up drinking the smoothie.” “It would be nice if there was a lounge that provided food.”
	Timing and Location	“Gosh, I would have said 10–15 minutes. Just long enough to get away and eat, catch my breath and go back.” “I take micro breaks. I’m a micro breaker. And maybe if you add it all together it equals a half hour break, but I mean, if I have to pee, I’ll find a good time, go pee for 3 minutes. Go get coffee for 7 minutes, black out on my desk for 3 minutes.” “Maybe 10 minutes if you’re going to use the bathroom or eat, and if you’re pumping, like 20–30 minutes.” “I’d rather have multiple small breaks.” “Not to speak for the group, but it seems like the consensus is that we all think everyone should eat, a small break is fine... I think when it gets long, I mean I think a 30-minute break, although, everyone is probably DUE a 30-minute break ...it’s excessive in our line of work.”
